# Removal of lead ions (Pb^2+^) from water and wastewater: a review on the low-cost adsorbents

**DOI:** 10.1007/s13201-022-01703-6

**Published:** 2022-06-22

**Authors:** Imran Rahman Chowdhury, Shakhawat Chowdhury, Mohammad Abu Jafar Mazumder, Amir Al-Ahmed

**Affiliations:** 1 Department of Civil and Environmental Engineering, King Fahd University of Petroleum and Minerals, Dhahran, 31261 Saudi Arabia; 2 Department of Chemistry, King Fahd University of Petroleum and Minerals, Dhahran, 31261 Saudi Arabia; 3 Interdisciplinary Research Center for Construction and Building Materials, King Fahd University of Petroleum and Minerals, Dhahran, 31261 Saudi Arabia; 4 Interdisciplinary Research Center for Advanced Materials, King Fahd University of Petroleum and Minerals, Dhahran, 31261 Saudi Arabia; 5 Interdisciplinary Research Center for Renewable Energy and Power Systems, King Fahd University of Petroleum and Minerals, Dhahran, 31261 Saudi Arabia

**Keywords:** Water and wastewater treatment, Lead contamination, Health risks, Removal of lead ions, Low-cost adsorbents, Reuse and regeneration

## Abstract

The presence of lead compounds in the environment is an issue. In particular, supply water consumption has been reported to be a significant source of human exposure to lead compounds, which can pose an elevated risk to humans. Due to its toxicity, the International Agency for Research on Cancer and the US Environmental Protection Agency (USEPA) have classified lead (Pb) and its compounds as probable human carcinogens. The European Community Directive and World Health Organization have set the maximum acceptable lead limits in tap water as 10 µg/L. The USEPA has a guideline value of 15 µg/L in drinking water. Removal of lead ions from water and wastewater is of great importance from regulatory and health perspectives. To date, several hundred publications have been reported on the removal of lead ions from an aqueous solution. This study reviewed the research findings on the low-cost removal of lead ions using different types of adsorbents. The research achievements to date and the limitations were investigated. Different types of adsorbents were compared with respect to adsorption capacity, removal performances, sorbent dose, optimum pH, temperature, initial concentration, and contact time. The best adsorbents and the scopes of improvements were identified. The adsorption capacity of natural materials, industrial byproducts, agricultural waste, forest waste, and biotechnology-based adsorbents were in the ranges of 0.8–333.3 mg/g, 2.5–524.0 mg/g, 0.7–2079 mg/g, 0.4–769.2 mg/g, and 7.6–526.0 mg/g, respectively. The removal efficiency for these adsorbents was in the range of 13.6–100%. Future research to improve these adsorbents might assist in developing low-cost adsorbents for mass-scale applications.

## Introduction

Heavy metals in water can pose risks to human and ecological health. Lead is one of the toxic heavy metals that can pose risks due to exposure from the aquatic and air media (Wani et al. 2015). It is one of the major pollutants responsible for soil, water, and atmospheric pollution, which is harmful to aquatic and human life even at a low concentration (Blanco et al. 2021). Lead can affect almost every organ and system in the human body. In particular, children aged below 6 years are most sensitive to the effects of lead exposure. Low concentrations of lead in children's blood can cause hearing and learning problems, anemia, behavior anomalies, slowed growth, lower intelligence quotient, and hyperactivity (Wani et al. 2015). During pregnancy, lead is released from bones as maternal calcium and helps develop the fetus's bones (Wani et al. 2015). It can also cross the placental barrier exposing the fetus to lead poisoning, resulting in severe effects on the mother and the developing fetus, including reduced fetus growth and premature birth (Charkiewicz and Backstrand 2020; Wani et al. 2015). Adults exposed to lead can suffer from cardiovascular effects, increased blood pressure and incidence of hypertension, decreased kidney function, and reproductive problems (Charkiewicz and Backstrand 2020; Wani et al. 2015). Due to its toxicity, the International Agency for Research on Cancer (IARC) and US Environmental Protection Agency (USEPA) have classified it as a probable human carcinogen (USEPA 2004; WHO 2006). Health Canada has set the maximum acceptable concentration (MAC) of lead in drinking water as 5 µg/L based on as low as reasonably achievable (ALARA) (Health Canada 2020). The European Community Directive and World Health Organization (WHO) have set the maximum acceptable lead limits in tap water as 10 µg/L (Hayes and Hoekstra 2010; WHO 2011). The USEPA has an action level of 15 µg/L in drinking water (USEPA 2009).

Lead occurs as lead sulfide or complex ore of lead and zinc sulfide in nature (Acharya 2013; Meena et al. 2020). Lead and its byproducts are released into the soil, air, and aquatic environments due to different industrial activities such as manufacturing industries of matches, explosives, pigments, photographic materials, printing, storage batteries, television tube, and paint industries (Kumar et al. 2020). It is also released into the environment with automobile emissions, sewage discharge, combustion of fossil fuel, urban and agricultural runoff, forest fires, volcanic eruptions, etc. (Cabral-Pinto et al. 2020; Cabral-Pinto and Ferreira da Silva 2019; Kumar et al. 2020). It can reach groundwater or surface water through industrial and domestic wastewater discharged into the water bodies or from acidic rain leached to the soils. In drinking water, the lead piping system is one of the primary sources of lead contamination.

Removal of lead ions from drinking water and wastewater is important for source protection and safe water supplies. A few hundred publications were reported on the removal of lead ions. The lead removal methodologies can be broadly categorized into adsorption, chemical precipitation, electrochemical reduction, ion exchange, liquid membrane separation, cementation, and solvent extraction (Abdullah et al. 2019; Azimi et al. 2017). Among these methods, adsorption has been reported to be the most popular process for its application feasibility and higher efficiency. The commonly used commercial adsorbents are zeolites, activated alumina, silica gel, and synthetic polymers (Baimenov et al. 2020; Delgado et al. 2018; Dlamini et al. 2020; Renu et al. 2017). In recent years, nanoparticles and carbon nanotubes (CNTs) have been used as adsorbents for removing heavy metals from water and wastewater (Fiyadh et al. 2019; Xu et al. 2018). The greater pore diameter and pore volume increase the adsorption capacity of the CNTs (Koh and Cheng 2014). Most commercial and CNT-based adsorbents are expensive, and the regeneration of these adsorbents is often not feasible.

**Fig. 1:**
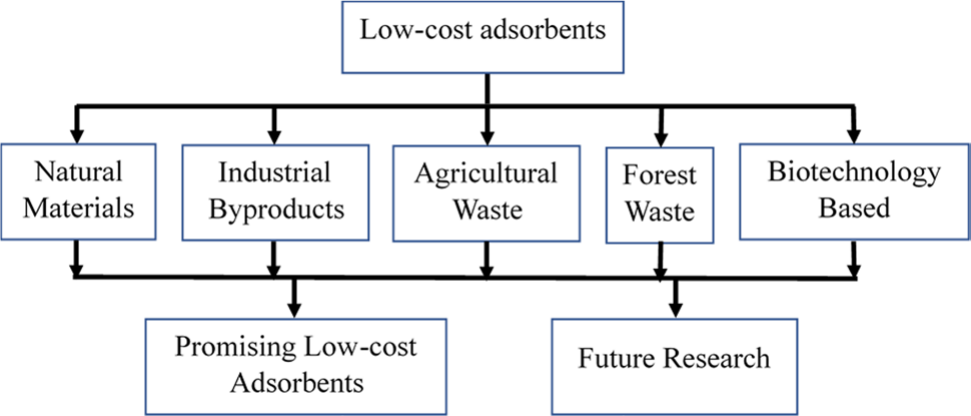
A schematic of the study process

There is a need to develop inexpensive adsorbents to remove lead ions from water and wastewater. In recent years, many studies have focused on activated carbon (AC)-based functionalized adsorbents produced from different materials, including domestic and industrial byproducts, polymers, and agricultural waste materials. Many of these adsorbents are likely to be inexpensive and efficient. This study reviewed the low-cost adsorbents following the methodology presented in Fig. 1. Low-cost adsorbents can be defined as the adsorbents that are inexpensive, require easy processing, available and abundant in nature. Based on the sources, the low-cost adsorbents were broadly categorized into five groups: natural materials, industrial byproducts, agricultural waste, forest waste, and biotechnology-based materials. The adsorption capacity and lead removal efficiency of these adsorbents was investigated. The advantages and limitations of these adsorbents were noted and compared. The scopes of improvements of the promising adsorbents were identified, and future research needs were highlighted.

## Technologies for lead removal

The most common methods for removing lead ions from water and wastewater include precipitation, coagulation-flocculation, ion exchange, adsorption, and membrane separation. Precipitation and coagulation-flocculation techniques produce large amounts of sludge (Crini and Lichtfouse 2019). In addition, these techniques are unlikely to reduce the lead ions to below the guideline value. Ion-exchange is expensive and requires pretreatment for wastewater as the exchanger matrices get clogged by the organics in the wastewater (Chowdhury et al. 2016; Yuan and Wood 2018). Membrane distillation (MD), a thermally driven membrane separation process, offered high removal of heavy metals from highly concentrated solutions (Lou et al. 2020; Alkhudhiri et al. 2020). The maximum removal of lead ions was 98% using the air gap MD process (Alkhudhiri et al. 2020). In addition, MD effectively removed coexisting metal ions from an aqueous solution (Lou et al. 2020; Alkhudhiri et al. 2020). The limitations of membrane separation include membrane fouling, low membrane durability, high operating cost, high equipment cost, and low permeation flux (Anis et al. 2019). The techniques mentioned above have their own set of benefits as well. However, the present study aims to review the adsorption technology using low-cost adsorbents. Hence, the discussion in the following sections will be limited to the low-cost adsorbents.

### Adsorption

Adsorption has been reported to be the most commonly applied technique for the removal of lead and other heavy metal ions from water and wastewater. The adsorption techniques often follow different types of equilibrium models. Some of the equilibrium equations for metal adsorption from an aqueous solution are shown in Table 1. Among the equilibrium models, the Langmuir and Freundlich isotherms are widely used for metal ion adsorption. The Langmuir adsorption isotherm model depicts the formation of monolayer metal ions on the outer surfaces of adsorbents with limited adsorption sites. The Freundlich isotherm model is empirical that represents the relationships between solute concentration on the adsorbent surface and solute concentration in the liquid, assuming a heterogeneous adsorbent surface. The adsorption equilibrium is attained when the rate of adsorption of the metal ions on a surface is equal to the desorption rate of the same metal ions. Adsorption techniques are very efficient, whereas the others have intrinsic limitations such as the production of a large amount of sludge, low efficiency, critical operating conditions, and expensive disposal (Renu et al. 2017). In addition, low-cost materials can be directly used as adsorbents or to prepare adsorbents to reduce the cost. To better assess the progress in developing the adsorbents, the low-cost adsorbents were classified into five categories: natural materials, industrial byproducts, agricultural waste, forest waste, and biotechnology-based materials. The source materials are widely available in nature and/or disposed of as waste, indicating that these materials have a great potential to develop low-cost adsorbents and are presented below:

**Table 1:** Types of adsorption equations

Isotherm	Equation
Single component dsorption	$q = \frac{{(C_{o} - C_{f} )V}}{m}$
Langmuir isotherm	$q_{e} = \frac{{q_{m} K_{L} C_{e} }}{{1 + K_{L} C_{e} }}$
Brunauer-Emmett eller (BET) isotherm	$\frac{1}{{W\left( {\frac{{P_{0} }}{P} - 1} \right)}} = \frac{1}{{ W_{m} C}} + \frac{{\left( {C - 1} \right)}}{{ W_{m} C}}\frac{{P_{0} }}{P}$
Freundlich isotherm	$q_{e} = K_{F} C_{e}^{\frac{1}{n}}$
Gibbs isotherm	$d\gamma = - \sum \left( {\frac{{n_{i} }}{A}} \right)d\mu_{i}$
Temkin Isotherm	$q_{e} = \left( {\frac{{{\text{RT}}}}{{B_{T} }}} \right){\text{ln}}\left( {A_{T} C_{e} } \right)$
Dubinin-Radushkevich DRK) isotherm	$q_{e} = q_{s} {\text{exp}}( - K_{{{\text{ad}}}} \varepsilon^{2} )$

Here *q* = the metal concentration retained in the sorbent phase (mg/g); *C*_0_ = the initial concentrations of the metal ion in solution (mol/l); *C*_*f*_ = the initial and final concentrations of the metal ion in solution (mol/l); *V* = the solution volume (liters); *m* = the mass of sorbent (g); *q*_*e*_ = the quantity of metal adsorbed on the surface of the adsorbent (mg/g); *C*_*e*_ = the amount of metal present in the solution at equilibrium condition (mg/L); *q*_*m*_ = the maximum adsorption capacity of the adsorbent (mg/g); *K*_*L*_ = the Langmuir constant related to energy of adsorption (L/mg); *K*_*F*_ = the Freundlich constant; *W* = weight of gas adsorbed; *P*/*P*_0_ = relative pressure; *W*_*m*_ = weight of adsorbate as monolayer; *C* = BET constant; *γ* = interfacial tension; (*n*_*i*_/*A*) = the number of moles of component adsorbed per unit area; *μ*_*i*_ = the chemical potential of the surfactant solution; *A*_*T*_ = Temkin isotherm equilibrium binding constant (L/g); *B*_*T*_ = Temkin isotherm constant; R = universal gas constant (8.314 J/mol/K); *T* = Temperature (K); *qs* = theoretical isotherm saturation capacity (mg/g); *K*_ad_ = Dubinin–Radushkevich isotherm constant (mol^2^/kJ^2^) and *ε* = Dubinin–Radushkevich isotherm constant

#### Natural materials

The natural materials used for the removal of lead ions from aqueous solution include natural clay (Khalfa et al. 2021), sand particles (Lai et al. 2000), peat moss (Lubbad and Al-Batta 2020), natural goethite (Abdus Salam and Adekola 2005), natural bentonite clay (Pfeifer et al. 2020), chitin (Kim et al. 2006) and talc surface (Chandra et al. 2005). The natural bentonite clay and acid-activated bentonite clay were reported to have the maximum adsorption capacity of 83.0 and 92.9 mg/g of lead ions, respectively (Budsaereechai et al. 2012). Ghahremani et al. (2021) impregnated activated carbon nanoparticles on the surface of the expanded lightweight clay aggregates and used them as adsorbents for Pb^2+^ removal. The maximum adsorption capacity was 22.8 mg/g, which removed 99% of Pb^2+^ from a concentration of 100 mg/L with an adsorbent dose of 10 g/L. The Aloji clay activated with HCl had a BET surface area of 214.8 m^2^/g and an adsorption capacity of 333.3 mg/g. It removed up to 97.3% of Pb^2+^ for the concentration of 30–150 mg/L (Obayomi and Outa 2019). The maximum zeolite and bentonite adsorption capacities were 137.0 and 119.7 mg/g, respectively (Pfeifer et al. 2020). Zeolite was effective for pH in the range of 3–7, while the optimal pH for bentonite was in the range of 7–8.5. The initial concentrations of Pb^2+^ were in the range of 362.6–2693.6 mg/L (Pfeifer et al. 2020).

Using the Saudi Arabian clay, Al-Jlil and Alsewailem reported the maximum adsorption of Pb^2+^ of 30 mg/g. The natural goethite removed almost 100% of Pb^2+^ for a concentration up to 750 mg/L (Abdus Salam and Adekola 2005). The point of zero charges of natural goethite was 7.8, which was greater than the experimental value of pH. Although the adsorption of positive ions like Pb^2+^ was not favorable under this condition, the adsorption might take place on the negatively charged surface sites. The amount of Pb^2+^ adsorbed on the goethite surface was a function of the metal ion affinity for the goethite surface and the type of surface complex formed during adsorption. The adsorption sites of the natural goethite were composed of three different OH^−^ coordination groups (Abdus Salam and Adekola 2005). The adsorption process showed a steady increase with time to achieve equilibrium. When it reached equilibrium, no significant uptake of metal ions occurred, representing the general characteristics of metal ion adsorption on iron oxide (Arora 2019; Sherlala et al. 2018). Katoh et al. (2016) used apatite and performed Pb^2+^ removal experiments in the presence and absence of organic acid. The maximum removal capacity was 1330, 1010, 896, and 782 mg/g in the presence of formic acid, no acid, malic acid, and citric acid, respectively (Katoh et al. 2016). The primary mechanism of Pb^2+^ removal by apatite/hydroxyapatite was dissolution and subsequent precipitation of lead phosphate/hydroxyphosphate such as pyromorphite (Kaludjerovic-Radoicic and Raicevic 2010; Oliva et al. 2012, 2010). There might be other mechanisms such as ion exchange and surface complexation. In the absence of organic acid, the Pb^2+^ were removed by the precipitation of lead phosphate minerals in apatite. Besides, the removal mainly occurred on the surface of apatite particles (Katoh et al. 2016). In the presence of formic acid, there was an increase in apatite dissolution due to the complexation between formic acid and Pb^2+^ in the solution. As a result, hydrogen ions from the formic acid disassociated, and the pH of the solution decreased, which caused an increase in Pb^2+^ removal capacity (Katoh et al. 2016). Rashed (2001) reported the maximum adsorption capacity of Pb^2+^ of 99 mg/g using chalcopyrite as an adsorbent. It removed up to 100% Pb^2+^ for the initial concentration of 50 mg/L with a contact time of 96 h (Rashed 2001). The maximum removal capacity of peat moss (Akinbiyi 2000) and sphagnum peat moss (Ratoi et al. 2008) was reported to be 96% and 98%, respectively. Table 2 summarizes the natural material-derived adsorbents used for Pb^2+^ removal. The maximum adsorption capacity was in the range of 0.8–333.3 mg/g (Table 2). The natural and phosphorylated chitin adsorbents showed the highest adsorption capacity (264 and 258 mg/g, respectively) for the initial concentrations of 100–300 mg/L and 50 mg/L, respectively (Table 2). The agbani clay showed the lowest adsorption capacity (0.8 mg/g) for the initial concentrations in the range of 20–100 mg/L (Dawodu et al. 2012). The lead removal performances were reported to be higher than 90% for several natural material-derived adsorbents (Table 2).

**Table 2:** Natural materials as adsorbents

Sl. No	Adsorbent	Maximum sorption apacity (mg/g)	Maximum removal (%)	Optimum pH	Dose (g/L)	Concentration (mg/L)	Temperature (°C)	Remarks	References
1	Natural goethite	5.0	100.0	3.0–5.0	40.0	5.0–750.0	27.0	Low-cost	Abdus Salam and Adekola (2005)
2	Natural clays honeycomb monoliths	2.5	> 90.0	4.5	0.6–200.0	10.0–200.0	25.0	Low-cost	Ahrouch et al. (2019)
3	Peat moss	48.2	96.0	5.5–6.0	0.04–0.2	10.0	RT	Low-cost	Akinbiyi (2000)
4	Saudi Arabian Clay	30.0	–	4.3	20.0	50.0–500.0	20.0	Low-cost	Al-Jlil and Alsewailem (2009)
5	Dijah-Monkin bentonite clay	8.7	–	5.1	1.0	10.0–50.0	25.0–65.0	Low-cost	Alexander et al. (2018)
6	Acid activated bentonite clay	92.9	–	–	20.0	100.0–5000.0	RT	Low-cost	Budsaereechai et al. (2012)
7	Natural bentonite clay	83.0	–	–	20.0	100.0–5000.0	RT	Low-cost	
8	Talc surface	8.0	> 98.0	6.0	1.0–50.0	5.0–500.0	20.0–70.0	Low-cost	Chandra et al. (2005)
9	Agbani clay	0.8	–	6.0	100.0	20.0–100.0	45.0	Low-cost	Dawodu et al. (2012)
10	Carbon nanoparticle impregnated on clay aggregate	22.8	99.0	6.0	6.0–10.0	10.0–500.0	25.0–55.0	Low-cost	Ghahremani et al. (2021)
11	Natural clay material	35.2	68.0	6.0		50.0–150.0	RT	Low-cost	Khalfa et al. (2021)
12	Chitin natural	264.0	–	4.0	10.0	100.0–300.0	15.0–35.0	Low-cost	Kim et al. (2006)
13	Chitin, phosphorylated	258.0	–	4.0	10.0	100.0–300.0	15.0–35.0	–	
14	Iron-coated sand	–	100.0	2.5–6.5	50.0	10.4–20.7	10.0–60.0	–	Lai et al. (2000)
15	Sphagnum peat moss	9.5	97.6	7.0	10.0–30.0	1.0–100.0	25.0	Low-cost	Lubbad and Al-Batta (2020)
16	Lignite	61.4	91.7	5.0	0.4–4.0	15.0–175.0	20.0	Low-cost	Mlayah et al. (2021)
17	Activated Aloji clay	333.3	97.3	7.0	1.0–5.0	30.0–150.0	30.0–50.0	Low-cost	Obayomi and Auta (2019)
18	Bentonite	119.7	98.1	3.0–7.0	8.0	362.6–2693.6	25.0	Low-cost	Pfeifer et al. (2020)
19	Zeolite	137.0	99.5	7.0–8.5	8.0	362.6–2693.6	25.0	Low-cost	
20	Barite	32.0	90.0	7.0–9.0	5.0–40.0	50.0–1000.0	RT	Low-cost	Rashed (2001)
21	Chalcopyrite	99.0	100.0	7.0–9.0	5.0–40.0	50.0–1000.0	RT	Low-cost	
22	Talc	44.0	78.0	7.0–9.0	5.0–40.0	50.0–1000.0	RT	Low-cost	
23	Sphagnum peat moss	67.1	98.0	6.0	1.25–10	34.0–507.0	RT	Low-cost	Ratoi et al. (2008)
24	Natural sand particles	24.9	91.5	6.0	25.0	0.05–5.0	RT	Low-cost	Shawket et al. (2011)
25	Seaweed, brown	1.4		4.0	0.5–2.0	6.2–677.5	30.0	Low-cost	Vieira et al. (2007)
26	Shanghai silty clay	26.5	100.0	6.0	4.0–40.0	10.0–300.0	25.0	Low-cost	Wang and Zhang (2021)
27	Calcined bentonite clay	94.0	90.9	5.0	0.5	5.0–200.0	20.0–60.0	Low-cost	Zbair et al. (2019)
28	Seaweed Ascophyllum nodosum	117.3	–	4.0	44.0	10.0	RT	–	Zhang and Banks (2006)
29	Sphagnum moss immobilized	32.5	–	4.0	44.0	10.0	RT	–	

*RT* Room temperature

Among the natural material-based adsorbents, activated Aloji clay showed the highest adsorption capacity (333.3 mg/g) (Obayomi and Auta 2019). The maximum removal of activated Aloji clay was 97.3% for a 30–150 mg/L concentration range. The natural chitin also had a high adsorption capacity (264 mg/g) (Kim et al. 2006).

#### Industrial byproducts

Table 3 summarizes the findings of the industrial byproduct-based adsorbents in removing Pb^2+^ from the aqueous solution. The major industrial byproducts used for removing heavy metals include iron slag (Zhang et al. 2020a, b), steel slag (Pfeifer et al. 2020), fly ash (Gupta and Ali 2004; Sridevi et al. 2013; Woolard et al. 2000), sawdust (Krishnan et al. 2003; Taty-Costodes et al. 2003; Yu et al. 2001), red mud (Cabral-Pinto et al. 2020; Ghorbani et al. 2013), and blast furnace slag (Nguyen et al. 2018). The red mud, an industrial waste produced during bauxite ore processing, was reported to remove Pb^2+^ completely within 60 min of contact time for the initial concentration of 50 mg/L (Ghorbani et al. 2013). The adsorbent was effective over a wide range of pH (2–8). The acid treatment of red mud increased the adsorption capacity from 16.4 to 19.2 mg/g (Narayanan et al. 2018), which increased the removal efficiency from 79.6 to 85.2%. Further coating with iron oxide increased the adsorption capacity and removal efficiency to 27.0 mg/g and 93.6%, respectively (Narayanan et al. 2018). Following acid treatment and iron oxide coating, the adsorbent’s surface became rougher, and the structure became more porous, leading to increased adsorption capacity (Narayanan et al. 2018). The steel dust, an industrial byproduct generated from the steel industry, was used to remove Pb^2+^ from the aqueous solution. The maximum adsorption capacity of Ladle Furnace and Cyclone steel dust were 208.9 and 39.8 mg/g, respectively (Bouabidi et al. 2018).

**Table 3:** Industrial by-products as adsorbents

Sl. No	Adsorbent	Maximum sorption capacity (mg/g)	Maximum removal (%)	Optimum pH	Dose (g/L)	Concentration (mg/L)	Temperature (°C)	Remarks	References
1	Sawdust	–	99.0	6.5	5.0–40.0	25.0	25.0	Low-cost	Abdel-Ghani et al. (2007)
2	Meranti tree sawdust	37.2	90.0	7.0	10.0–80.0	25.0–250.0	25.0	Low-cost	Ahmad et al. (2009)
3	Carbonized sawdust	87.7	–	8.0	0.5–5.0	100.0–600.0	25.0–50.0	Low-cost	Aigbe and Kavaz (2021)
4	Zinc oxide nanoparticle-carbonized sawdust matrix	92.6	–	8.0	0.5–5.0	100.0–600.0	25.0–50.0	Low-cost	
5	Olive stone waste	22.4	99.3	5.0	3.0	20.0	30.0	Low-cost	Alslaibi et al. (2014)
6	Modified coal fly ash	31.4	–	3.0	20.0	10.0–1000.0	27.0	–	Astuti et al. (2020)
7	Ladle Furnace steel dust	208.9	–	4.0–5.0	2.0	20.0–200.0	25.0	Low-cost	Bouabidi et al. (2018)
8	Cyclone steel dust	39.8	–	4.0–5.0	2.0	20.0–200.0	25.0	Low-cost	
9	Red mud (bauxite ore processing waste)	–	100.0	2.0–8.0	10.0	50.0	25.0	Low-cost	Ghorbani et al. (2013)
10	Fly ash bagasse	2.5	95.0–96.0	6.0	2.0–16.0	5.0–70.0	30.0–50.0	Economic	Gupta and Ali (2004)
11	Teak sawdust	40.7	–	5.0	0.1–10.0	15.0–200.0	25.0 ± 2.0	Low-cost	Gupta et al. (2009)
12	Sand powder	9.9	–	7.0	20.0	200.0–2000.0	20.0–40.0	Low-cost	Jung et al. (2019)
13	Chitosan-coated sand powder	10.8	–	4.5	20.0	200.0–2000.0	20.0–40.0	Low-cost	
14	Sunflower wood waste fly ash	138.4	99.8	7.6	1.0–5.0	20.0–100.0	23.0	Low-cost	Kalak et al. (2021)
15	Sawdust activated carbon	109.8	98.9	6.5	2.0	51.8–414.4	30.0	Low-cost	Krishnan et al. (2003)
16	Fly ash	49.8	–	4.0–6.0	4.0	100.0.-1000.0	25.0	Low-cost	Liu et al. (2016)
17	Fly ash mixed with geopolymer	118.6	–	4.0–6.0	4.0	100.0.-1000.0	25.0	Low-cost	
18	Olive oil waste	22.8	–	5.0	10.0	10.0–1000.0	25.0	–	Martin-Lara et al. (2009)
19	Red Mud	16.4	79.6	5.0	1.0–8.0	10.0–100.0	RT	Low-cost	Narayanan et al. (2018)
20	Acid-treated red mud	19.2	85.2	7.0	1.0–8.0	10.0–100.0	RT	Low-cost	
21	Iron oxide-coated acid-treated red mud	27.0	93.6	6.0	1.0–8.0	10.0–100.0	RT	Low-cost	
22	Blast furnace slag	4.9	80.0	6.0–7.0	0.1–20.0	5.0	25.0	Low-cost	Nguyen et al. (2018)
23	Fly ash	3.4	90.0	6.0–7.0	0.1–20.0	5.0	25.0	–	
24	Sago waste activated carbon	524.0	67.0	5.0	25.0–100.0	75.0–175.0	RT	–	Nordin et al. (2020)
25	Fly ash-containing geopolymer monoliths	6.3	68.0	5.0–7.0	–	50.0	RT	Low-cost	Novais et al. (2016)
26	Waste beer yeast	55.7	96.4	1.0–5.0	0.5–40.0	25.0–100.0	30.0	Low-cost	Parvathi (2007)
27	Steel slag	59.8	85.6	7.0	8.0	362.6–2693.6	25.0	Low-cost	Pfeifer et al. (2020)
28	Meranti sawdust	34.3	97.0	6.0	5.0	1.0–200.0	30.0	Low-cost	Rafatullah et al. (2009)
29	Low-grade manganeseore	142.9	–	4.5	1.0–6.0	50.0–500.0	27.0	Low-cost	Rout et al. (2009)
30	Sawdust	18.0	70.9	4.9	20.0	414.4–621.6	30.0	Low-cost	Shukla and Roshan (2005)
31	Sawdust activated carbon	93.4	90.1	5.0	2.0	51.8–414.4	30.0	Low-cost	Sreejalekshmi et al. (2009)
32	Coal fly ash	–	91.7	–	0.5–1.5	100.0	RT	–	Sridevi et al. (2013)
33	Sawdust of Pinus sylvestris	22.2	98.0	5.5	1.0–10.0	1.0–50.0	25.0	Low-cost	Taty-Costodes et al. (2003)
34	Modified fly ash	352.2	–	5.0	8.0	500–2000	25.0	Low-cost	Woolard et al. (2000)
35	Sawdust	–	98.8	2.0–5.0	20.0–50.0	10.0–200.0	25.0–60.0	Low-cost	Yu et al. (2001)
36	Chitosan	8.3	–	6.0	10.0	10.0–200.0	30.0	Low-cost	Zulkali et al. (2006)

*RT* Room temperature

A column of fly ash bagasse was used as an adsorbent to remove Pb^2+^ with an initial concentration of 5.0–70.0 mg/L. The 0.5 mL/min flow rate achieved a removal efficiency of 95–96% (Gupta and Ali 2004). Using the coal fly ash, Sridevi et al. reported 90.4% removal of Pb^2+^ for an initial concentration of 100 mg/L (Sridevi et al. 2013). The blast furnace slag and fly ash's maximum adsorption capacity was 4.9 and 3.4 mg/g, respectively (Nguyen et al. 2018). Woolard et al. (2000) reported that the modified fly ash could remove up to 352.2 mg/g of Pb^2+^. The simultaneous adsorption experiments were performed using the modified coal fly ash (MCFA) to remove Pb^2+^ and Zn^2+^ (Astuti et al. 2020). The maximum adsorption capacity of Pb^2+^ and Zn^2+^ ions were 31.4 and 27.0 mg/g, respectively, and the effective pH was 3.0. The point of zero charges of the adsorbent was 12.15, implying that adsorption of Pb^2+^ might be done in a solution with a pH lower than 12. However, Pb^2+^ are removed by precipitation rather than adsorption at a pH higher than 9 (Kavand et al. 2020). The Pb^2+^ ions had a stronger electronegativity and a lower hydrated ionic radius than Zn^2+^, leading to greater adsorption affinity to attach with a functional group in the MCFA through electrostatic attraction (Xiong et al. 2019). The maximum adsorption capacity of fly ash mixed with geopolymer was nearly 2.5 times (118.6 mg/g) the adsorption capacity of fly ash (49.8 mg/g) (Liu et al. 2016). The geopolymers had similar adsorption mechanisms as faujasite or zeolite. As geopolymers are zeolite analogs, geopolymer technology can be an energy-saving, low-cost, and environmentally friendly process in adsorbent manufacturing. Novais et al. (2016) used fly ash containing geopolymeric monoliths in packed beds that could be conveniently collected when exhausted, which has a significant benefit over powdered adsorbents. Furthermore, the manufacturing process reuses the biomass fly-ash, which reduces the environmental effects of waste disposal and the costs of adsorbent production.

The sawdust-derived activated carbon removed 90–99% of Pb^2+^ from a solution of 51.8–414.4 mg/L (Krishnan et al. 2003; Sreejalekshmi et al. 2009). The maximum adsorption capacity of the industrial byproduct-based adsorbents in removing Pb^2+^ was in the range of 2.5–524.0 mg/g (Table 3). The sawdust activated carbon showed the highest adsorption capacity of 109.8 mg/g for the initial concentration of 51.8–414.4 mg/L (Krishnan et al. 2003). The surface properties, such as particle size, ash content, apparent density, cation-exchange capacity, total acidic sites, and carboxylic acid content of sawdust activated carbon was 0.096 mm, 5.31%, 1.02 g/mL, 3.16 meq/g, 4.02 meq/g, and 1.95 meq/g, respectively (Sreejalekshmi et al. 2009). The value of the point of zero charges was reported to be 5.3. At pH of less than 5.3, the predominant lead species were Pb^2+^ ions while the other species [e.g., Pb(OH)^+^, Pb_2_(OH)^3+^, Pb_3_(OH)_4_^2+^ and Pb_4_(OH)_4_^4+^] were present in small quantities (Sreejalekshmi et al. 2009). The maximum adsorption capacity of zinc oxide nanoparticles (ZnOnp), carbonized sawdust (CSD), and ZnOnp-CSD matrix were 70.4, 87.7, and 92.6 mg/g, respectively (Aigbe and Kavaz 2021). Zinc oxide nanoparticles have been reported as an efficient and low-cost adsorbent having a high surface area and high metal removal capacity (Kumar et al. 2013). The ZnOnp-CSD matrix is likely to be a low-cost adsorbent. However, the adsorption capacity was nearly similar to the CSD (Aigbe and Kavaz 2021). The findings indicated that several studies on the industrial byproducts had more than 90% removal of Pb^2+^ from aqueous solution (Table 3).

Comparing the maximum adsorption capacity, the sago waste-activated carbon performed the best among the industrial byproducts. The adsorption capacity was 524 mg/g (Nordin et al. 2020). The adsorption capacity of modified fly ash was reported to be 352.2 mg/g (Woolard et al. 2000).

#### Agricultural waste

Table 4 summarizes the findings of the agricultural waste-based adsorbents in removing lead ions from water and wastewater. The agricultural waste-based products were likely to be the low-cost adsorbents as these are the discarded items mainly, abundant, and easy to use. Several functional groups in the agricultural waste include hydrocarbons, carbohydrates, cellulose and hemicelluloses, starch, lignin, lipids, and proteins (Dai et al. 2018). These functional groups often maximize lead removal by bonding with the carboxylic (–COOH) groups following acid treatment (Aziz et al. 2019). Different types of agricultural waste were used to remove heavy metal ions (Table 4). The carboxylated jute stick-derived activated carbon's maximum adsorption capacity for Pb^2+^ was reported to be 2079 mg/g (Aziz et al. 2019). Several studies used the rice husk-based adsorbents in removing Pb^2+^ ions (Abdel-Ghani et al. 2007; Amen et al. 2020; Arabahmadi and Ghorbani 2017; Feng et al. 2004; Fooladgar et al. 2019; Gupta et al. 2009; Janyasuthiwong et al. 2015; Kamari et al. 2019; Mahmoud et al. 2020; Masoumi et al. 2016; Masoumi et al. 2016; Naiya et al. 2009; Nnaji et al. 2017; Shi et al. 2019; Sun et al. 2019; Wang et al. 2018; Zulkali et al. 2006), which showed the maximum adsorption capacity in the range of 5.7–1665.0 mg/g (Table 4). The rice husk ash showed an adsorption capacity of 91.7 mg/g, and the maximum removal efficiency was 99.3% (Naiya et al. 2009). Abdel-Ghani et al. (2007) used rice husk-activated carbon and attained 99% removal of Pb^2+^ with an initial concentration of 25 mg/L. Fooladgar et al. (2019) applied chitosan/rice husk ash/nano-γ alumina and reported a maximum adsorption capacity of 181.8 mg/g. The maximum removal was 91% with an initial concentration of 30 mg/L, which was achieved within 105 min. After the 6th cycle of regeneration, the adsorption capacity was more than 70% (Fooladgar et al. 2019).

**Table 4:** Agricultural waste as adsorbents

Sl. No	Adsorbent	Maximum sorption capacity (mg/g)	Maximum removal (%)	Optimum pH	Dose (g/L)	Concentration (mg/L)	Temperature (°C)	Remarks	References
1	Maize cobs	–	99.0	6.5	5.0–40.0	25.0	25.0	Low-cost	Abdel-Ghani et al. (2007)
2	Rice husks	–	99.0	6.5	5.0–40.0	25.0	25.0	Low-cost	
3	Plant powder	–	80.0	6.0	2.0	4.0–120.0	RT	–	Abdel-Halim et al. (2003)
4	Banana peels	66.7	100.0	5.5	0.1–1.0	10.0–100.0	RT	Low-cost	Afolabi et al. (2021)
5	Tea waste	73.0	96.0	5.0	0.5–40.0	5.0–100.0	30.0	Low-cost	Ahluwalia and Goyal (2005)
6	Rice husk biochar	–	96.4	5.5	1.0–4.0	1950.0	RT	Low-cost	Amen et al. (2020)
7	Wheat straw biochar	–	95.4	5.5	1.0–4.0	1950.0	RT	Low-cost	
8	Corncob biochar	–	96.9	5.5	1.0–4.0	1950.0	RT	Low-cost	
9	Peels of banana	2.2	85.3	5.0	10.0–90.0	30.0–80.0	25.0	Low-cost	Anwar et al. (2010)
10	Polythiophene-coated rice husk ash nanocomposite	34.5	98.1	4.0	5.0–20.0	50.0–400.0	25.0–65.0	–	Arabahmadi and Ghorbani (2017)
11	Carboxylated jute stick-derived activated carbon	2079.0	99.8	4.0–7.0	1.0	5.0–500.0	15.0–27.0	Low-cost	Aziz et al. (2019) and Chowdhury et al. (2020)
12	Lentil husk	81.4	98.0	5.0	2.0	20.0–250.0	20.0–35.0	Low-cost	Basu et al. (2015)
13	Coffee residue activated with zinc chloride	63.3	75.0	5.8	1.0	10.0–90.0	25.0	–	Boudrahem et al. (2011)
14	Wheat bran	87.0	–	4.0–7.0	5.0–60.0	50.0–1000.0	20.0–60.0	–	Bulut and Baysal (2006)
15	Walnut shell	9.9	92.3	4.0	1.0–50.0	100.0	25.0	Low-cost	Çelebi and Gök (2017)
16	Peanut Hull-g-Methyl Methacrylate	370.4	99.3	5.7	2.0–12.0	5.0–100.0	20.0–50.0	–	Chaduka et al. (2020)
17	Modified peanut shells	130.5	–	4.6–5.0	–	4144.0	RT	–	Chamarthy et al. (2001)
18	Chemically modified moso bamboo	181.8	85.0	5.0	0.5–4.0	200.0	25.0–45.0	–	Chen et al. (2020)
19	Arca shell	18.3	98.6	4.0	0.1–15.0	10.0–500.0	25.0 ± 2.0	–	Dahiya et al. (2008)
20	Olive cake	19.5	92.3	6.0	20.0	50.0–1000.0	20.0–35.0	–	Doyurum and Celik (2006)
21	Pomegranate peel	13.9	65.0	5.6	0.25	10.0–50.0	26.0 ± 1.0	Low-cost	El-Ashtoukhy et al. (2008)
22	Pomegranate peel activated carbon	14.0	80.0	5.6–7.6	2.5	10.0–50.0	26.0 ± 1.0	Low-cost	
23	Pomegranate peel chemically treated	18.0	90.0	5.6–7.6	2.5	10.0–50.0	26.0 ± 1.0	–	
24	Peanut hull hydrochar activated by H_3_PO_4_	162.1	–	–	–	10.0–700.0	RT	Cost-effective	Fang et al. (2017)
25	Peanut hull hydrochar activated by KOH	158.0	–	–	–	10.0–700.0	RT	Cost-effective	
26	Ash of rice husk	12.6	–	5.6–5.8	2.0	40.0	15.0–30.0	Low-cost	Feng et al. (2004)
27	Olivestone waste	9.3	80.0 ± 2.0	5.5	13.3	41.4–3108.0	20.0	Low-cost	Fiol et al. (2006)
28	Chitosan/rice husk ash/nano-γ alumina	181.8	91.0	5.0	–	250.0–550.0	10.0–40.0	–	Fooladgar et al. (2019)
29	Soya bean	0.7	80.0	4.0	10.0–40.0	1240.0	28.0–40.0	Low-cost	Gaur et al. (2018)
30	Tea waste	–	92.8 ± 1.4	7.0	–	100.0	28.0–42.0	Low-cost	Ghaffar (2008)
31	Coconut shell granular activated carbon	21.9	–	5.0	2.0	5.0–70.0	37.0 ± 2.0	–	Goel et al. (2005)
32	Seed hull of the palm tree	3.8	–	4.0	120.0	100.0–500.0	30–60.0	–	Gueu et al. (2007)
33	Coconut	4.4	–	4.0	120.0	100.0–500.0	30–60.0	–	
34	Peanut hulls	69.8	–	5.0	0.1–10.0	15.0–200.0	25.0 ± 2.0	Low-cost	Gupta et al. (2009)
35	Discarded tea leaves	35.9	–	5.0	0.1–10.0	15.0–200.0	25.0 ± 2.0	Low-cost	
36	Peels of banana	72.8	–	5.0	0.1–10.0	15.0–200.0	25.0 ± 2.0	Low-cost	
37	Rice husk	31.1	–	5.0	0.1–10.0	15.0–200.0	25.0 ± 2.0	Low-cost	
38	Rice stem	49.6	–	5.0	0.1–10.0	15.0–200.0	25.0 ± 2.0	Low-cost	
39	Coir fibers	52.0	–	5.0	0.1–10.0	15.0–200.0	25.0 ± 2.0	–	
40	Okra waste	5.7	99.0	5.0	10.0–40.0	25.0–100.0	25.0	Low-cost	Hashem (2007)
41	Palm kernel fiber	47.6	99.2	5.0	1.5–5.0	120.0	36.0 ± 4.0	–	Ho and Ofomaja (2006)
42	Hazelnut husks	13.1	97.2	5.7	2.0–20.0	5.0–200.0	18.0	Low-cost	Imamoglu and Tekir (2008)
43	Palm shell	95.2	–	5.0	5.0	10.0–700.0	27.0	–	Issabayeva et al. (2008)
45	Palm kernel husk	–	88.0	5.0	20.0–100.0	5.0–15.0	RT	–	Iyagba and Opete (2009)
46	Palm kernel shell	–	81.0	5.0	20.0–100.0	5.0–15.0	RT	–	
47	Groundnut shell	–	98.0	3.0	2.4–8.8	5.0–105.0	25.0	Low-cost	Janyasuthiwong et al. (2015)
48	Orange peel	–	99.0	5.0	2.4–8.8	5.0–105.0	25.0	Low-cost	
49	Rice husk	–	85.0	3.0	2.4–8.8	5.0–105.0	25.0	Low-cost	
50	Sunflower wood waste fly ash	138.4	99.8	–	2.0–5.0	20.0–100.0	23.0	Low-cost	Kalak et al. (2021)
51	Rice husk nanocomposite	1665.0	96.8	5.2	0.1–2	15.0–150.0	RT	Low-cost	Kamari et al. (2019)
52	Sugarcane bagasse chemically modified	189.0	–	5.0–6.0	1.0	200.0–400.0	RT	–	Karnitz et al. (2007)
53	Walnut shell	–	96.2	6.0–10.0	–	30.0	RT	Low-cost	Kazemipour et al. (2008)
54	Almond	–	99.8	6.0–10.0	–	30.0	RT	–	
55	Apricot stone	–	89.6	6.0–10.0	–	30.0	RT	–	
56	Hazelnut shell	–	96.9	6.0–10.0	–	30.0	RT	Low-cost	
57	Pistachio shell	–	83.0	6.0–10.0	–	30.0	RT	Low-cost	
58	Activated bamboo charcoal	53.8	83.0	5.0	1.0–5.0	50.0–90.0	29.0	Low-cost	Lalhruaitluanga et al. (2010)
59	Raw bamboo charcoals	10.7	13.6	5.0	1.0–5.0	50.0–90.0	29.0	Low-cost	
60	Orange peel xanthate	204.5	–	5.0	5.0	10.0–100.0	30.0	–	Liang et al. (2009)
61	Orange peel formaldehyde-treated	46.6	99.0	5.0	10.0	30.0–250.0	RT	–	Lugo-Lugo et al. (2009)
62	EDTA functionalized bamboo activated carbon	123.5	–	5.0–6.0	0.8	25.0–250.0	20.0–60.0	–	Lv et al. (2018)
63	Bamboo activated carbon	45.5	–	5.0–6.0	0.8	25.0–250.0	20.0–60.0	–	
64	Functionalized graphene from rice husk	748.5	99.8	7.0	10.0–50.0	20.7	18.0–80.0	Low-cost	Mahmoud et al. (2020)
65	Coffee endocarp waste	174.4	57.7	–	1.0	300.0	RT	Low-cost	Mariana et al. (2021)
66	Coffee endocarp waste treated with HCl	193.0	63.9	–	1.0	300.0	RT	–	
67	Coffee endocarp waste treated with NaOH	272.6	89.9	–	1.0	300.0	RT	–	
68	Grape stalk	49.7	–	5.5	6.7	198.9	20.0	–	Martinez et al. (2006)
69	Treated rice husk	93.5	95.0	7.0	5.0	100.0–800.0	20.0–50.0	Low-cost	Masoumi et al. (2016)
70	Mustard husk	30.5	100.0	6.0	6.0–12.0	1.0–5.0	20.0–60.0	Low-cost	Meena et al. (2008)
71	Cocoa shells	33.4	95.0	2.0	15.0	100.0	22.0	–	Meunier et al. (2003)
72	Corn stover biochar	25.0	98.0	6.0	2.5	5.0–250.0	RT	Low-cost	
73	Orange peel biochar	11.1	96.0	6.0	2.5	5.0–250.0	RT	Low-cost	
74	Pistachio biochar	2.5	35.0	5.0	2.5	5.0–250.0	RT	Low-cost	
75	Ash of rice husk	91.7	99.3	5.0	5.0	3.0–100.0	30.0	Low-cost	Naiya et al. (2009)
76	Chemically modified rose petals	118.4	90.0	5.0	1.0	10.0–640.0	30.0	–	Nasir et al. (2007)
77	Rice husk ash	26.1	80.0	3.0	–	10.0–130.0	30.0–40.0	Low-cost	Nnaji et al. (2017)
78	Capsicum annuum seeds	38.7	90.0	5.0	0.4–6.0	100.0	20.0–40.0	–	Özcan et al. (2007)
79	Acid-treated wheat bran	79.4	82.8	6.0	2.0	50.0–500.0	25.0–60.0	–	Ozer (2007)
80	Ponkan peel	112.1	–	5.0	8.0	0.5–1000.0	25.0	–	Pavan et al. (2008)
81	Almond	8.1	68.0	6.0–7.0	6.3–25.0	20.7–207.2	25.0 ± 1.0	–	Pehlivan et al. (2009)
82	Shells of hazelnut	28.2	90.0	6.0–7.0	6.3–25.0	20.7–207.2	25.0 ± 1.0	Low-cost	
83	Ceiba pentandra hulls	25.5	99.5	6.0	1.0–4.0	40.0–200.0	30.0 ± 1.0	–	Rao et al. (2008)
84	Apricot stone	1.3	95.3	7.0	10.0–40.0	5.0–500.0	RT	–	Rashed (2006)
85	Peach stone	2.3	97.6	7.0	10.0–40.0	5.0–500.0	RT	–	
86	Nitric acid activated Caryota urens seeds carbon	42.9	89.0	7.0	0.5–5.0	50.0–250.0	30.0–60.0	–	Ravulapalli and Kunta (2018)
87	Onion skins	200.0	93.5	6.0	0.75	25.0–200.0	30.0	Low-cost	Saka et al. (2011)
88	Citrus peels original and protonated	658.9	90.0	5.0	1.0	20.0–400.0	21.0–25.0	–	Schiewer and Balaria (2009)
89	Coconut shell activated carbon	26.5	92.5	4.5	0.2–2.0	10.0–50.0	35.0–45.0	–	Sekar et al. (2004)
90	Pretreated bamboo biochar	181.2	–	3.0–4.5	0.8	50.0–400.0	30.0	–	Shen et al. (2021)
91	Rice husk biochar	26.7	84.5	6.0	5.0	20.7–621.6	RT	–	Shi et al. (2019)
92	Coir	26.3	87.0	4.9	20.0	116.0–651.4	30.0	–	Shukla and Roshan (2005)
93	Shells of groundnut	22.0	82.8	4.9	20.0	116.0–651.4	30.0	Low-cost	
94	Jute	18.6	73.4	4.9	20.0	116.0–651.4	30.0	Low-cost	
95	Maize bran	142.9	96.8	6.5	20.0	100.0–150.0	20.0–40.0	–	Singh et al. (2006)
96	Magnetic rice husk biochar	148.0	95.0	2.5–5.8	2.5	10.0–500.0	25.0	–	Sun et al. (2019)
97	Corncobs chemically modified	43.4	–	5.0	4.0	20.7–414.4	25.0	–	Tan et al. (2010)
98	Corncobs native	16.6	–	5.0	4.0	20.7–414.4	25.0	–	
99	Horticultural peat	36.5	96.0	4.5–7.0	10.0	100.0–600.0	25	–	Ulmanu et al. (2008)
100	Pecan nutshell	196.1	–	5.5	1.0–15.0	10–1000.0	25.0	–	Vaghetti et al. (2009)
101	Magnetic rice husk biochar	129.0	91.7	7.0	0.02–1	1.00–80.0	25.0	Cost-effective	Wang et al. (2018)
102	Antep pistachio	27.1	95.1	3.5	2.5–20.0	5.0–100.0	30.0–60.0	–	Yetilmezsoy and Demirel (2008)
103	Palm shell polyethyleneimine-impregnated	53.5	–	5.0	5.0	20.0–750.0	25.0	–	Yin et al. (2008)
104	Sun flower waste	33.2	–	4.0	–	10.0	RT	Low-cost	Zhang and Banks (2006)
105	Plant maize	2.3	–	4.0	–	10.0	RT	–	
106	Dehydrated banana peels biochar	359.0	> 90.0	7.0	0.25–5.0	5.0–1000.0	RT	Low-cost	Zhou et al. (2017)
107	Fresh banana peels biochar	193.0	> 90.0	7.0	0.25–5.0	5.0–1000.0	RT	Low-cost	
108	Rice husk	5.7	–	5.0	2.0–20.0	10.0–200.0	30.0–60.0	Low-cost	Zulkali et al. (2006)

*RT* Room temperature

The surface areas of rice husk biochar (RHBC), wheat straw biochar (WSBC), and corncob biochar (CCBC) were 255.8, 24.5, and 9.0 m^2^/g, respectively (Amen et al. 2020). The total pore volume was 0.245, 0.0251, and 0.0015 cm^3^/g, respectively. The contact periods for adsorption experiments were varied from 15 to 120 min. The removal efficiency of RHBC, WSBC, and CCBC in the ranges of 78.5–96.4%, 82.6–95.4%, and 85.6–96.9%, respectively (Amen et al. 2020). The magnetic rice husk biochar reported the maximum adsorption capacity of 148 and 129 mg/g, respectively (Sun et al. 2019; Wang et al. 2018). A low-cost amine-functionalized nanocomposite adsorbent was prepared by extracting amorphous silica from rice husk (Kamari et al. 2019). The adsorbent's surface area and pore volumes were 695 m^2^/g and 0.65 cm^3^/g, respectively. The adsorbent had a maximum adsorption capacity of 1665 mg/g. The maximum removal efficiency was 96.8%. In addition, the adsorption capacity decreased only 10% after the 5^th^ cycle of regeneration (Kamari et al. 2019). In another work, rice husk was used to synthesize graphene quantum dots (GQDOs) with 2D morphology and further chemically modified with Ba(OH)_2_ to increase the number of surface hydroxyl groups (GQDOs-Ba) (Mahmoud et al. 2020). The GQDOs were listed as low-cost and low-toxicity compounds. The GQDOs-Ba had high thermal stability (50 and 800 °C). The maximum adsorption capacity and removal efficiency of GQDOS-Ba was 748.5 mg/g and 99.8%, respectively (Mahmoud et al. 2020). The equilibrium was reached within 15 s while the samples were microwaved under constant temperature (Mahmoud et al. 2020).

The maximum uptake capacity of hazelnut husk and mustard husks was 13.1 and 30.5 mg/g, respectively (Imamoglu and Tekir 2008; Meena et al. 2008). The black gram husk had an uptake capacity of 50.0 mg/g (Saeed et al. 2005). Gupta et al. (2009) used peanut hulls, discarded tea leaves, banana peels, rice husk, rice stem, and coir fibers in removing lead ions. The maximum adsorption capacity was reported to be in the range of 31.1–72.8 mg/g (Table 4). The orange peel xanthate was reported to adsorb up to 204.5 mg/g of Pb^2+^ (Liang et al. 2009). The formaldehyde-treated orange peel was reported to have a maximum adsorption capacity of 46.6 mg/g (Lugo-Lugo et al. 2009). The peels of banana (Afolabi et al. 2021; Anwar et al. 2010; Gupta et al. 2009), pomegranate (El-Ashtoukhya et al. 2008), citrus (Schiewer and Balaria 2009), and skins of onions (Saka et al. 2011) were also studied in removing Pb^2+^ from water. The maximum uptake capacity of banana peel was 2.2 mg/g, and the maximum removal efficiency was 85.3% (Anwar et al. 2010). The adsorption capacity of banana peel was 66.7 mg/g (Afolabi et al. 2021). The pH_pzc_ of banana peel was 4.83, indicating that the surface of the adsorbent was acidic and favorable for cation adsorption. It was reported to remove 98–100% Pb^2+^ from 10 mg/L solutions within 4 h. The adsorbent was also tested in binary metal ion systems with Cu^2+^, which showed that the adsorption of Pb^2+^ was higher than copper (Afolabi et al. 2021). The ionic radius of Pb^2+^ is smaller than Cu^2+^. As such, more Pb^2+^ were adsorbed readily on the active sites of banana peels. Zhou et al. (2017) used hydrothermal carbonization (HTC) to prepare dehydrated and fresh banana peels biochar. HTC has been considered a low-cost carbonization technique to produce effective biochar adsorbents with simple procedures and low energy consumption (Zhou et al. 2017). The dehydrated and fresh banana peel biochars had a maximum adsorption capacity of 359 and 193 mg/g, respectively (Zhou et al. 2017). The maximum uptake capacity and removal efficiency for the onion skin was 200 mg/g and 93%, respectively (Saka et al. 2011). Using the citrus peel, the maximum uptake capacity and removal efficiencies were reported to be 658.9 mg/g and 90%, respectively (Schiewer and Balaria 2009).

The activated carbon produced from hazelnut, pistachio, and almond had higher surface areas (Dolas et al. 2011). The Brunauer–Emmett–Teller (BET) surface area, Dubinin–Radushkevich (DR) surface area, and DR micropore volume of the activated carbon produced from pistachio shells, treated with ZnCl_2_ and HCl, and activated at 900 °C were 3256 m^2^/g, 3822 m^2^/g, and 1.36 cm^3^/g, respectively (Dolas et al. 2011). When treated with sodium chloride and activated at 900 °C, these values were 3895 m^2^/g, 5235 m^2^/g, and 1.86 cm^3^/g (Dolas et al. 2011). Several studies used coconut and coconut shell-derived activated carbon, which showed the maximum adsorption capacity in the range of 3.8–26.5 mg/g (Goel et al. 2005; Gueu et al. 2007; Sekar et al. 2004). A number of studies used the shells of peanut (Chaduka et al. 2020; Fang et al. 2017; Gupta et al. 2009), walnut (Çelebi and Gök, 2017; Kazemipour et al. 2008), almond (Kazemipour et al. 2008; Pehlivan et al. 2009), hazelnut (Kazemipour et al. 2008; Pehlivan et al. 2009), palm (Issabayeva et al. 2008; Iyagba and Opete 2009), arca (Dahiya et al. 2008), pistachio (Kazemipour et al. 2008; Meunier et al. 2003), and cocoa (Meunier et al. 2003) in removing Pb^2+^ from aqueous solution, which showed the maximum adsorption capacity in the range of 2.5–370.4 mg/g. The removal efficiency of walnut shells, hazelnut shell, and pistachio shell was 96.2, 96.9, and 83%, respectively (Kazemipour et al. 2008). Another study showed that the removal efficiency of groundnut shells was 98% (Janyasuthiwong et al. 2015). Çelebi and Gök (2017) used a walnut shell and removed 92.3% lead from an initial concentration of 100 mg/L Pb^2+^ solutions. The maximum adsorption capacity of peanut hulls was 69.8 mg/g (Gupta et al. 2009). Cahduka et al. (2020) prepared a novel graft copolymer by copolymerizing activated carbon from peanut hulls and methyl methacrylate and reported the maximum adsorption capacity of 370.4 mg/g. The adsorbent removed 99.3% of Pb^2+^ from an initial concentration of 76.25 mg/L within one hour with an adsorbent dose of 4.5 g/L (Chaduka et al. 2020). The palm shell had the maximum uptake capacity of 92.6 mg/g, and after pretreating, the uptake capacity was increased to 95.2 mg/g (Issabayeva et al. 2008). The point of zero charges of palm shell activated carbon was 1.43 (Issabayeva et al. 2006). In addition, a high concentration of acidic surface groups was present in the palm shell-activated carbon, which promoted higher adsorption of metal ions at higher pH (Saka et al. 2012).

The acid or alkaline treatment of agricultural waste-derived activated carbon typically increased the adsorption capacity (Aziz et al. 2019). The maximum adsorption capacity of coffee endocarp waste, coffee endocarp waste treated with HCl, coffee endocarp waste treated with NaOH were 174.4, 193.0, and 272.6 mg/g, respectively (Mariana et al. 2021). The chemical activation increased the adsorption capacity and the removal efficiency. The chemical activation released the impurities on the adsorbent resulting in the widening of the pores and promoting the formation of functional groups that effectively absorb the metal ions (Mariana et al. 2021). In addition, the NaOH-activated sorbent had the largest surface area to pore volume ratio and the largest pore size, which might be the cause of increased adsorption capacity. The acid-treated wheat bran showed a maximum adsorption capacity of 79.4 mg/g at pH of 6 and an initial concentration of 50–500 mg/L (Ozer 2007). However, an earlier study by Bulut and Baysal showed the maximum adsorption capacity of 87.0 mg/g for the untreated wheat bran for a wide range of pH (4–7) and higher initial concentrations (50–1000 mg/L) (Bulut and Baysal 2006). Singh et al. (2006) reported the maximum adsorption capacity of the maize bran-based activated carbon of 142.9 mg/g. The Pb^2+^ removal efficiency was 96.8% for the initial concentration of 100 mg/L at a pH of 6.5. Boudrahem et al. (2011) used coffee residue as the raw materials for powder-activated carbon, which was activated ZnCl_2_. The pore surface area and micropore volume of the activated carbon were 890 m^2^/g and 0.77 cm^3^/g, respectively. With an initial concentration of 10–90 mg/L Pb^2+^, the maximum uptake capacity of the activated carbon was 63.3 mg/g (Boudrahem et al. 2011). Some other agricultural wastes, including okra waste (Hashem 2007), sunflower waste (Zhang and Banks 2006), and grape stalk (Martinez et al. 2006), were used in removing Pb^2+^ from aqueous solution. The okra waste removed 99% of Pb^2+^ for an initial concentration of 240 mg/L of lead solution (Hashem 2007).

The maximum uptake capacity of raw bamboo charcoal was 10.7 mg/g (Lalhruaitluanga et al. 2010). Following activation by chemical treatment, the maximum uptake capacity was increased to 53.8 mg/g (Lalhruaitluanga et al. 2010). The maximum removal efficiency was also increased from 13.6 to 83.0% (Lalhruaitluanga et al. 2010). The adsorption capacity of ethylene diamine tetraacetic acid (EDTA) functionalized bamboo activated carbon (123.5 mg/g) was more than twice the adsorption capacity of raw bamboo activated carbon (45.5 mg/g) (Lv et al. 2018). The adsorption capacity of chemically modified Moso bamboo with pyromellitic dianhydride was (181.8 mg/g) (Chen et al. 2020), which was almost similar to the adsorption capacity of ammonium persulfate pretreated bamboo biochar (181.2 mg/g) (Shen et al. 2021). These studies indicated that modifying or pretreating bamboo-activated carbon significantly increased the adsorption capability of the adsorbent. However, the increase in cost due to modification or pretreatment was not discussed. The Pb^2+^ removal performances were reported to be higher than 90% for a large number of agricultural waste-based activated carbon (Table 4). Further details on the agricultural waste-based adsorbents can be found in Table 4.

The adsorption capacity of carboxylated jute stick activated carbon (Chowdhury et al. 2020) and rice husk nanocomposite (Kamari et al. 2019) was very high. The carboxylated jute stick activated carbon, and rice husk nanocomposite adsorption capacity was 2079 mg/g and 1665 mg/g, respectively. Both the adsorbents are likely to be low-cost.

#### Forest waste

The forest waste is likely to be closely linked to agricultural byproducts. However, as these materials are not the direct byproducts of agricultural activities, the forest waste-based adsorbents are separately discussed in this study. The natural processes of shedding tree leaves and barks have made forests the abundant sources of low-cost and environment-friendly raw materials for the adsorbents (Bhattacharyya and Sharma 2004; Khatoon et al. 2018). Several past studies have investigated the forest waste-based adsorbents in removing heavy metals from wastewater. The maximum adsorption capacity of the forest waste-derived adsorbents ranged from 0.4 to 769.2 mg/g (Table 5). Using the Viscum album leaves, Erenturk and Malkoc (2007) reported the maximum adsorption capacity and removal efficiency of 769.2 mg/g and 92.2%, respectively, for an initial concentration of 100–500 mg/L. Iqbal et al. (2009) reported 99.1% removal of Pb^2+^ using the mango peel waste. Gupta et al. (2009) reported the maximum adsorption capacity of 31.5 mg/g using the mango leaves. Argun and Dursun (2007) reported 90.0% removal of Pb^2+^ using the Pinus nigra tree bark for an initial concentration of 35 mg/L, while the maximum adsorption capacity was 49.0 mg/g. The maximum adsorption capacity of Ficus religiosa leaves was 37.5 mg/g (Qaiser et al. 2009). The maximum adsorption capacity of Peepul tree leaves was 127.3 mg/g (Gupta et al. 2009).

**Table 5:** Forest waste as adsorbents

Sl. No	Adsorbent	Maximum sorption capacity (mg/g)	Maximum removal (%)		Optimum pH	Dose (g/L)	Concentration (mg/L)	Temperature (°C)	Remarks	References
1	Nile rose plant	–		98.7	8.5	5.0–40.0	5.0–50.0	25.0	–	Abdel-Ghani and El-Chaghaby (2007)
2	Leaves, Casuarina glauca tree	–		97.4	6.5	20.0	5.0–50.0	25.0	Low-cost	Abdel-Ghani et al. (2008)
3	Citrus limetta leaves	69.8		99.5	6.0	0.25–1.5	5.0–100.0	25.0	–	Aboli et al. (2020)
4	Streblus asper leaves	3.1		71.9	8.0	20	100.0	25.0	–	Adebayo et al. (2012)
5	Pinus nigra tree bark	49.0		90.0	8.0	2.5	35.0	RT	Low-cost	Argun and Dursun (2007)
6	Carpobrotus edulis	175.6		98.0	6.0	25.0	100.0	25.0	Low-cost	Benhima et al. (2008)
7	Euphorbia echinus	165.1		–	6.0	25.0	100.0	25.0	Low-cost	
8	Launaea arborescens	129.9		–	6.0	25.0	100.0	25.0	Low-cost	
9	Senecio anteuphorbium	149.6		98.0	6.0	25.0	100.0	25.0	Low-cost	
10	Leaf powder Azadirachta indica (neem)	300.0		93.0	7.0	0.2–1.2	50.0–150.0	27.0	Low-cost	Bhattacharyya and Sharma (2004)
11	Leaves bael	104.0		85.0	5.1	0.2–10.0	48.2–180.2	30.0–50.0	–	Chakravarty et al. (2010)
12	Azadirachta indica (neem leaves)	39.7		93.5	7.0	1.0–20.0	50.0–300.0	25.0–45.0	Low-cost	Elkhaleefa et al. (2021)
13	Viscum album leaves	769.2		92.2	3.0	0.1–0.8	100–500.0	25.0–55.0	Low-cost	Erenturk and Malkoc (2007)
14	Hickory hydrochar activated by KOH	135.7		–	–	–	10.0–700.0	RT	Cost-effective	Fang et al. (2017)
15	Peepul tree leaves	127.3		–	5.0	0.1–10.0	15.0–200.0	25.0 ± 2.0	Low-cost	Gupta et al. (2009)
16	Mango tree leaves	31.5		–	5.0	0.1–10.0	15.0–200.0	25.0 ± 2.0	Low-cost	
17	Grass clippings	29.1		–	5.0	0.1–10.0	15.0–200.0	25.0 ± 2.0	Low-cost	
18	Cereal chaff	12.5		–	5.5	8.0	8.0–96.0	20.0	–	Han et al. (2005)
19	Leaf powder Hevea brasiliensis	46.7		–	5.0	1.0–20.0	10.0–200.0	30.0	Low-cost	Hanafiah et al. (2006)
20	Tree fern	38.1		–	4.9	4.0	200.0	20.0	Low-cost	Ho (2005)
21	Ficus benghalensis	12.3		52.2	5.5	–	20.0–100.0	RT	Low-cost	Hymavathi and Prabhakar (2019)
22	Mango peel waste	99.1		–	5.0	2.5	10.0–600.0	25.0 ± 2.0	Low-cost	Iqbal et al. (2009)
23	Bamboo dust	2.2		70.1	7.2	10.0–28.0	600.0	30.0 ± 1.0	–	Kannan and Veemaraj (2009)
24	C. demersum	44.8		–	5.0–6.0	2.0	2.0–64.0	25.0	–	Keskinkan et al. (2007)
25	M. spicatum	46.5		–	5.0–6.0	2.0	2.0–64.0	25.0	–	
26	Schleichera oleosa bark	69.4		97.0	6.0	10.0	10.0–100.0	30.0–50.0	Low-cost	Khatoon et al. (2018)
27	Tamarix leaves activated carbon	42.2		97.9	6.0	0.25–3.0	10.0–100.0	25.0–55.0	Low-cost	Koohzad et al. (2019)
28	Bael tree leaf	4.1		90.1	5.0	5.0–30.0	25.0–100.0	30.0	Low-cost	Kumar and Gayathri (2009)
29	Lawny grass modified	321.2		100.0	5.0–5.8	1.7	103.6–414.4	5.0–55.0	Low-cost	Lu et al. (2009)
30	Curry leaf powder	60.9		92.0	6.5	1.0–4.0	50.0–200.0	25.0	Low-cost	Mukherjee et al. (2020)
31	Eucalyptus camaldulensis Dehn. Bark	184.4		–	5.0	4.0	20.7–2072.0	25.0–60.0	Low-cost	Patnukao et al. (2008)
32	Leaves Ficus religiosa	37.5		–	4.0	0.5	100.0	20.0–40.0	Low-cost	Qaiser et al. (2009)
33	Ziziphus jojoba	80.0		–	6.0	1.0–30.0	20.0–700.0	20.0–50.0	Feasible	Salman et al. (2019)
34	Eriobotrya Japonica leaves	73.1		–	6.0	1.0–30.0	20.0–700.0	20.0–50.0	Feasible	
35	Ulva lactuca	34.7		–	5.0	2.0–40.0	10.0–400.0	20.0–50.0	–	Sari and Tuzen (2008)
36	Prosopis cineraria leaf ash	0.4		100.0	6.0	1.0–3.0	30.0–120.0	25.0	–	Shahmaleki et al. (2020)
37	Cephalosporium aphidicola	92.3		62.5	1.0–6.0	0.4–3.0	100.0.–400.0	20.0–40.0	–	Tunali et al. (2006)
38	Tobacco leaves	238.6		–	2.0	0.5–3.0	5.0–50.0	30.0–70.0	Low-cost	Yogeshwaran and Priya (2021)
39	Natural condensed tannin	114.9		91.0	4.2	1.0	100.0–1000.0	20.0	–	Zhan and Zhao (2003)

*RT* Room temperature

The maximum adsorption capacity of curry leaf powder was 60.9 mg/g (Mukherjee et al. 2020). It had a surface area of 21.56 m^2^/g. The removal efficiency of curry leaf powder increased while pH was increased from 4.5 to 6.5. The adsorbent also showed less removal efficiency at a pH higher than 10.5 (Mukherjee et al. 2020). As the removal efficiency was directly related to the protonation or deprotonation of surface functional groups, the presence of more chelating sites at pH range 5.5–7.5 makes the adsorbent more efficient (Hojati and Landi 2015; He et al. 2018). The precipitation of metal hydroxides and their limited solubility at highly alkaline pH (pH > 10.5) might be the reason for less removal of metals (Kumar et al. 2010). The surface area of Tamarix leaves activated carbon was 252.3 m^2^/g, which had a maximum adsorption capacity of 42.2 mg/g (Koohzad et al. 2019). The highest removal efficiency for Pb^2+^ was 97.9%, achieved within 60 min at a temperature of 25 °C. The initial concentration of Pb^2+^ was 10 mg/L, and the adsorbent dosage was 3 g/L (Koohzad et al. 2019). The Azadirachta indica (neem leaves) showed similar adsorption capacity (39.7 mg/g) and removal efficiency (93.5%) (Elkhaleefa et al. 2021). Ziziphus jojoba and Eriobotrya Japonica leaves showed adsorption capacity of 80.0 mg/g and 73.1 mg/g, respectively (Salman et al. 2019). The maximum adsorption capacity of tobacco leaves was 238.6 mg/g. The optimum pH, temperature, contact time, and the adsorbent dose were 1.0, 30 °C, 80 min, and 0.5 g/L, respectively. The adsorption efficiency was 67.45% (Yogeshwaran and Priya 2021). These tree leaves are widely available in nature, which makes the adsorbent low-cost and environmentally friendly. The forest waste appeared to be an abundant natural resource for producing a mass scale of adsorbents. Further details on the forest waste-based adsorbents can be found in Table 5.

Among the forest waste-based adsorbents, Viscum album leaves showed a high adsorption capacity (769.2 mg/g) (Erenturk and Malkoc 2007). The removal efficiency was up to 92.2% for a concentration range of 100–500 mg/L.

#### Biotechnology-based materials

In recent years, biomass-based adsorbents have been used in removing heavy metal ions. Table 6 summarizes the application of biosorbents for the removal of lead ions from wastewater. The biochar obtained from pyrolysis of sludge is a low-cost, environmentally friendly material, which has the potential to be a heavy metal adsorbent. The maximum adsorption capacity of the biochar and activated biochar was 7.6 and 38.5 mg/g, respectively. After treating with hydrochloric acid (HCl), the adsorption capacity of the biochar and activated biochar was increased to 9.8 and 40.4 mg/g, respectively. After treating with hydrofluoric acid (HF), the adsorption capacity was further increased to 16.7 and 49.5 mg/g, respectively (Zhang et al. 2020a, b). The acid treatment increased the biochar's specific surface area and pore structure significantly. The specific surface area of activated biochar was increased from 583.4 to 718.7 and 991.6 m^2^/g after HCl and HF treatment, respectively (Zhang et al. 2020a, b). The surface areas were increased primarily because the inorganic minerals obstructing the pores of sludge-based biochar were washed away, revealing more pores and improving the pore structure characteristics. Further removal of silicon by hydrofluoric acid, which also blocked the pores, increased the adsorption capacity of biochar (Zhang et al. 2020a, b). Ho et al. (2017) used anaerobic digestion sludge biochar (ADSBC) and reported the maximum adsorption capacity of 53.4 mg/g at pH 6. Using the adsorbent dose of 10 g/L, up to 100% of lead ions were removed for an initial concentration of 100 mg/L (Ho et al. 2017). The ferric-activated biological sludge had the maximum uptake capacity of 43.0 mg/g, and it removed 98.5% of Pb^2+^ at the pH range of 4–6 (Yang et al. 2019).

**Table 6:** Biotechnology-based adsorbents

Sl. No	Adsorbent	Maximum sorption capacity (mg/g)	Maximum removal (%)	Optimum pH	Dose (g/L)	Concentration (mg/L)	Temperature (°C)	Remarks	References
1	Alginate-immobilized Chlorella vulgaris	–	> 90.0	6.0	3 × 10^7^ cells:1 ml	50.0	RT	Low-cost	Abdel-Hameed (2006)
2	Sewage sludge-derived biochar immobilized nanoscale zero-valent iron	–	91.0	4.0	1.5	15.0	RT	–	Diao et al. (2018)
3	Chlorella vulgaris	133.8	99.4	5.0	0.25–4.0	50.0	RT	Low-cost	Goher et al. (2016)
4	Biomass of Spirulina maxima	–	92.0	5.5	0.1–2.0	50.0	20.0	–	Gong et al. (2005)
5	Anaerobic digestion sludge biochar	53.4	100.0	6.0	1.0–10.0	100.0	20.0–40.0	–	Ho et al. (2017)
6	Aspergillus niger	172.2	45.5	4.0–5.4	–	200.0–1400.0	37.0	–	Iram et al. (2015)
7	Alkali-treated mango seed integuments	49.9	75.2	7.0	0.5–3.0	1.0–50.0	30.0	–	Kanjilal et al. (2014)
8	Neurospora crassa	43.3	–	4.0	2.0	5.0–300.0	25.0	–	Kiran et al. (2005)
9	Xanthan biopolymer integrated graphene oxide	199.2	80.8	5.2	0.1–1.0	10.0–300.0	30.0–70.0	–	Lai et al. (2020)
10	Phosphate-modified baker's yeast	92.0	88.2	5.0	0.2–2.0	25.0–250.0	25.0–40.0	Low-cost	Liu et al. (2018)
11	Sargassum glaucescens	244.5	–	5.0	–	207.2	20.0 ± 2.0	–	Naddafi et al. (2007)
12	Iron oxide modified clay-activated carbon composite beads	74.2	95.0	4.5	2.0	12.0–350.0	25.0	Low-cost	Pawar et al. (2018)
13	Calcium alginate beads doped Caryota urens seeds carbon	86.9	96.0	7.0	0.5–5.0	50.0–250.0	30.0–60.0	–	Ravulapalli and Kunta (2018)
14	Sodium alginate graft-poly(methyl methacrylate) beads	526.0	96.0	4.0	2.0	200.0–1000.0	RT	–	Salisu et al. (2016)
15	Encapsulated Agrobacterium fabrum	197.0	85.0	5.5	10 beads:2 ml	100.0–4000.0	37.0	–	Tiwari et al. (2017)
16	Bio-hybrid silsesquioxane/yeast	248.0	82.0	4.0	50–500 cm^3^/g	100.0–1000.0	25.0–40.0	Low-cost	Trama-Freitas et al. (2017)
17	Immobilized inactivated cells of Rhizopus oligosporus in calcium alginate	25.8	–	2.0–5.0	–	50.0–100	25.0	–	Xia et al. (2003)
18	Ferric-activated biological sludge	43.0	98.5	4.0–6.0	0.5–3.0	50.0	25.0	–	Yang et al. (2019)
19	Fungi Penicillium oxalicum	155.6	98.3	4.0–5.0	10^7^ spores/ml	100.0–2500.0	30.0	–	Ye et al. (2018)
20	Nano-ZnO/yeast composites	66.7	–	6.0	4.0	25.0–250.0	RT	Economical	Zhang et al. (2016)
21	Baker’s yeast	22.5	–	6.0	4.0	25.0–250.0	RT	Low-cost	
22	Sludge-based biochar	7.6	–	8.0	–	5.0–300.0	15.0–45.0	–	Zhang et al. (2020a, b)
23	Activated sludge-based biochar	38.5	–	8.0	–	5.0–300.0	15.0–45.0	–	
24	Sludge-based biochar pretreated with HCl	9.8	–	8.0	–	5.0–300.0	15.0–45.0	–	
25	Activated sludge-based biochar pretreated with HCl	40.4	–	8.0	–	5.0–300.0	15.0–45.0	–	
26	Sludge-based biochar pretreated with HF	16.7	–	8.0	–	5.0–300.0	15.0–45.0	–	
27	Activated sludge-based biochar pretreated with HF	49.5	–	8.0	–	5.0–300.0	15.0–45.0	–	

*RT* Room temperature

Chemical modification of yeast with silsesquioxanes by exploiting the reactivity of the nanostructures presented by silsesquioxanes positively affected the biosorption process in living cells (Trama-Freitas et al. 2017). It is a promising adsorbent at high concentrations (100–1000 mg/L) with excellent efficiency in short contact periods (15 min) at room temperature and pH of 4. The removal efficiency and maximum adsorption capacity were 82% and 248 mg/g, respectively (Trama-Freitas et al. 2017). The used yeast Saccharomyces cerevisiae is a waste of the alcoholic fermentation process. An inexpensive, readily available, and safe industrial microorganism, Baker’s yeast has been investigated to remove lead from aqueous solution (Liu et al. 2018; Zhang et al. 2016). Nano-ZnO/yeast composites had an adsorption capacity of 66.48 mg/g (Zhang et al. 2016). After adsorption/desorption for four cycles, it demonstrated more than 85% adsorption (Zhang et al. 2016), while the phosphate-modified baker's yeast (PMBY) exhibited more than 90% of the original adsorption capacity after four cycles of adsorption/desorption (92 mg/g) (Liu et al. 2018). Moreover, the equilibrium was reached within 3 min.

The maximum adsorption capacity of xanthan biopolymer integrated graphene composite was 199.2 mg/g (Lai et al. 2020). In addition, it retained 84.8% of its initial adsorption capacity after the 5th regeneration cycle indicating its high regenerable characteristics (Lai et al. 2020). The maximum uptake capacity of Penicillium chrysogenum was 155.6 mg/g (Ye et al. 2018). Goher et al. (2016) used Chlorella Vulgaris in alginate beads for removing Pb^2+^. The maximum uptake capacity and removal efficiency of Chlorella Vulgaris alginate (CVA) beads were 133.8 mg/g and 99.4%, respectively (Goher et al. 2016). Immobilization of Chlorella Vulgaris in alginate beads decreased the removal efficiency (Goher et al. 2016). The surface area and pore volume of CVA beads were 16.2 m^2^/g and 0.0116 cm^3^/g, respectively. The Fourier Transformation Infrared Spectroscopy (FTIR) analysis of CVA beads before and after Pb^2+^ showed a shift of the peak of different functional groups. This indicated the biosorption of Pb^2+^ with the cells. The shift of the peak, disappearance of peaks, and appearance of new peaks suggested that binding occurred on the surface of the CVA beads. However, up to 95% of Pb^2+^ were desorbed from the alginate beads using citric acid, which were reused at almost a similar efficiency (Goher et al. 2016). The maximum uptake capacity of the encapsulated agrobacterium fabrum was reported to be 197.0 mg/g at an optimum pH of 5.5. This study reported the maximum removal efficiency of 85% for the initial concentrations of Pb^2+^ in the range of 100–4000 mg/L (Tiwari et al. 2017). During the first 120 min, the adsorption rate was rapid, followed by a slower adsorption rate for up to 240 min prior to achieving equilibrium. The initial high adsorption rate indicated the highly porous structure of the composite beads. The intra-particle diffusion might cause a slower adsorption rate until it reaches equilibrium. The biosorbent showed good adsorption capacity even after repeated use for up to five consecutive cycles (Tiwari et al. 2017). Ravulapalli and Kunta (2018) developed activated carbon from the seeds of the Caryota urens plant (ACSCU) and impregnated ACSCU into the calcium alginate beads (CABCU). The ACSCU and CABCU removed 89% and 96% of lead ions, respectively, from an initial Pb^2+^ concentration of 50 mg/L with a dose of 2 g/L. The adsorption capacity of CABCU (86.9 mg/g) was almost double the adsorption capacity of ACSCU (42.9 mg/g). During 7 cycles of regeneration, the removal efficiency reduced from 96.0 to 80.7%, indicating the excellent reuse potential CABCU. The BET surface area and adsorption capacity of iron oxide-modified clay-activated carbon composite beads were 433 m^2^/g and 74.2 mg/g, respectively (Pawar et al. 2018). The adsorbent was also tested in removing low-level concentrations of toxic metal ions from a ternary mixture. It reduced Pb^2+^, Cd^2+^ and As^5+^ from 48.7, 52.3 and 51.2 µg/L to 1.21, 1.14, and 7.5 μg/L, respectively, which were below the WHO guidelines (Pawar et al. 2018).

The adsorption capacity of sodium alginate grafted poly(methyl methacrylate) beads (526 mg/g) was higher compared to other adsorbents (Salisu et al. 2016). The maximum removal efficiency was 96% for a 200–1000 mg/L concentration range. In addition, the beads were found to be regenerated multiple times.

## Promising low-cost adsorbents

Many natural material-derived adsorbents showed very good to excellent efficiency in removing lead ions. The natural sand particles removed 91.5% of Pb^2+^ from an aqueous solution, whereas the natural goethite removed up to 100% (Abdus Salam and Adekola 2005; Shawket et al. 2011). Among the adsorbents, peat moss, sphagnum peat moss, senecio anteuphorbium, acid-activated bentonite clay, activated aloji clay, bentonite, zeolite, barite, chalcopyrite, natural goethite, talc, chitin showed excellent performances (Table 2). The activated aloji clay had a maximum adsorption capacity of 333.3 mg/g. The maximum removal efficiency was 97.3%, while the concentrations of Pb^2+^ were varied from 30 to 150 mg/L. Bentonite and zeolite also showed excellent performance. The maximum adsorption capacity of bentonite and zeolite were 119.7 and 137.0 mg/g, respectively (Table 7). The maximum removal efficiency was 98.1% and 99.5%, respectively (Table 7). The cost of natural clay is $0.005-0.46/kg, and it is nearly 20 times cheaper than the commercial activated carbon (Babel and Kurniawan 2003). Although many natural material-based adsorbents showed very good to excellent performances, their application might face issues in terms of material availability, cost, environmental effects, and toxicity. The adsorbents are likely to produce large amounts of lead-containing sludge, which must be disposed of safely. Further, it is often challenging to desorb the lead ions from the adsorbents. Besides, the initial concentrations of lead ions were much higher in the laboratory experiments (Table 2), which were more reflective of industrial wastewater. The reported efficiency might not be similar for low concentrations of lead ions, such as surface water, groundwater, drinking water, and domestic wastewater. For application in drinking water, toxicity is an issue. The toxicity of these adsorbents is not well known.

**Table 7:** Promising low-cost adsorbents and their performances

SL No	Type	Adsorbent	Maximum sorption capacity (mg/g)	Maximum removal (%)	Advantage	Limitations	References
1	Natural material	Activated Aloji clay	333.3	97.3	Can be used over a wide range of pH and temperature	Limited field applications No information on reusability Production cost is unknown	Obayomi and Outa (2019)
2		Bentonite	119.7	98.1	Applicable for other heavy metals		Pfeifer et al. (2020)
3		Zeolite	137.0	99.5	Applicable for other heavy metals		
4	Industrial byproduct	Ladle Furnace steel dust	208.9		Applicable for industrial effluent	Toxicity data is not available Reproducibility should be investigated Cost information is not available	Bouabidi et al. (2018)
5		Sunflower wood waste fly ash	138.4	99.8	Applicable for other heavy metals		Kalak et al. (2021)
6		Fly ash mixed with geopolymer	118.6		Can be used over a wide range of pH and temperature		Liu et al. (2016)
7		Steel slag	59.8	85.6	Applicable for other heavy metals		Pfeifer et al. (2020)
9	Agricultural waste	Carboxylated jute stick-derived activated carbon	2079.0	99.8	Quick removal	Toxicity, reusability, and cost should be investigated Removal process depends on temperature	Aziz et al. (2019)
10		Lentil husk	81.4	98.0	Applicable for industrial effluent, easily desorbed		Basu et al. (2015)
11		Rice husk nanocomposite	1665.0	96.8	Regeneration without significant effect on efficiency		Kamari et al. (2019)
12		Functionalized graphene from rice husk	748.5	99.8	Applicable for industrial effluent		Mahmoud et al. (2020)
13		Coffee endocarp waste treated with NaOH	272.6	89.9	Can be applicable for other heavy metals		Mariana et al. (2021)
14		Formaldehyde-treated Onion skin	200.0	93.5	Can be used over a wide range of pH		Saka et al. (2011)
15		Magnetic rice husk biochar	129.0	91.7	Applicable for other heavy metals, recyclable		Wang et al (2018)
16		Dehydrated banana peels biochar	359.0	> 90.0	Can be used over a wide range of pH		Zhou et al (2017)
17		Fresh banana peels biochar	193.0	> 90.0			
18	Forest waste	Citrus limetta leaves	69.8	99.5	Applicable for other heavy metals	Limited field applications No information on reusability Production cost is unknown	Aboli et al (2020)
19		Carpobrotus edulis	175.6	98.0	Applicable for other heavy metals		Benhima et al (2008)
20		Leaf powder Azadirachta indica (neem)	300.0	93.0	Can be used over a wide range of pH		Bhattacharyya and Sharm (2004)
21		Viscum album leaves	769.2	92.2	Can be used over a wide range of temperature		Erenturk and Malko (2007)
22		Schleichera oleosa bark	69.4	97.0	Recyclable, can be used over a wide range of pH and temperature		Khatoon et al. (2008)
23		Natural condensed tannin	114.9	91.0	Favorable in lead removal from acidic wastewater		Zhan and Zhao (2003)
24	Biotechnology-based material	Phosphate-modified baker's yeast	92.0	88.2	Excellent regeneration capability	May not be feasible for drinking water applications Information on toxicity and health effects is not available	Liu et al. (2018)
25		Iron oxide modified clay-activated carbon composite beads	74.2	95.0	Applicable for other heavy metals		Pawar et al. (2018)
26		Bio-hybrid silsesquioxane/yeast	248.0	82.0	Quick removal		Trama-Freitas et al. (2017)

The most common industrial byproducts used as the adsorbents for lead removal are red mud, sunflower wood waste, blast furnace slag, sawdust, and fly ash. Many studies used sawdust, sawdust activated carbon, and sawdust waste as the adsorbents (Table 3). Among these, sawdust (Yu et al. 2001), sawdust activated carbon (Krishnan et al. 2003), meranti sawdust (Rafatullah et al. 2009), and sawdust of *Pinus sylvestris* (Taty-Costodes et al. 2003) showed the removal efficiency of 98.8, 98.9, 97.0, and 98.0%, respectively. In these studies, the corresponding initial concentrations were 10.–200, 51.8–414.4, 1–200, and 1–50 mg/L, respectively, and the pH was 2.0–5.0, 6.5, 6.0, and 5.5, respectively (Table 3). The average cost of wood residue from mills was reported to be US$0.018-0.036/Kg (Clauser et al. 2018). The sawdust waste might emerge as a potential low-cost raw material from the timber industries. The production cost of sawdust activated carbon was reported to be approximately US $7.0/kg (Krishnan et al. 2003). The beer yeast waste and coal fly ash removed 96.4 and 91.7% of Pb^2+^, respectively, from the 100 mg/L of the lead solution (Parvathi 2007; Sridevi et al. 2013). The beer yeast waste can be obtained at no cost, as the beer industries face problems in disposing of the waste (Parvathi 2007). Steel slag and steel dust also showed excellent performance in removing lead from water. The maximum adsorption capacity of ladle furnace steel dust was 208.9 mg/g. The maximum removal efficiency of steel slag was 85.6% (Table 3). The cost of steel slag in India was US$6.0-11.5/Kg (Dhoble and Ahmed 2018). Despite the low cost of the raw materials, the application of industrial byproducts might be limited to the wastewater only as the toxicity of the sawdust mix and beer yeast waste-based adsorbents are not well known.

The agricultural waste-based adsorbents comprised the most significant fraction of publications (Table 4). As demonstrated in Table 4, a wide variety of agricultural waste, including rice husk, orange peel, coconut, peanut, walnut, bran, coffee, tea waste, jute stick, and palm kernel shell, was used as the raw materials for adsorbents (Table 4). The rice husk-based adsorbents showed up to 99.8% removal of Pb^2+^. In the batch and column experiments, Naiya et al. (2009) showed the excellent performance of rice husk adsorbents. Up to 99.3% removal of Pb^2+^ was observed at a pH of 5.0, and the maximum adsorption capacity was 91.7 mg/g (Naiya et al. 2009). The rice husk nanocomposite and functionalized graphene from rice husk had the maximum adsorption capacity of 1665 and 748.5 mg/g, respectively (Table 7). The maximum removal efficiency was 99.8% and 96.8%, respectively (Table 7). The rice husk can be obtained free of cost or at a meager price as agricultural waste. The dry biomass of natural orange peel and okra waste also removed 99% of lead ions from an aqueous solution (Hashem 2007; Lugo-Lugo et al. 2009). The biochar of banana peels removed > 90% lead ions from 5 to 1000 mg/L lead solution (Zhou et al. 2017). The tea waste was reported to remove 96% of Pb^2+^ from 20 mg/L lead solution (Ahluwalia and Goyal 2005). At pH 6.5 and an initial concentration of 100 mg/L, the maize bran-based adsorbents removed 96.8% of Pb^2+^ with the maximum adsorption capacity of 142.9 mg/g (Singh et al. 2006). The onion skin and citrus peel also showed excellent results (Saka et al. 2011; Schiewer and Balaria 2009). Up to 99.8% removal of Pb^2+^ was achieved using the carboxylated jute stick-based activated carbon for the concentrations in the range of 5–500 mg/L (Aziz et al. 2019; Chowdhury et al. 2020). In this study, the maximum adsorption capacity was reported to be 2079 mg/g (Aziz et al. 2019; Chowdhury et al. 2020). Jute stick was an agricultural waste, which was cheap and eco-friendly. In addition to wastewater, this adsorbent might be used for drinking water systems following the toxicity assessment.

The forests were abundant natural resources of raw materials for adsorbents. The Schleichera oleosa bark had maximum adsorption capacity and maximum removal efficiency of 69.4 mg/g and 97%, respectively (Khatoon et al. 2018). The mango peel waste removed 98.8% Pb^2+^ with a maximum adsorption capacity of 99.1 mg/g (Iqbal et al. 2009). The Nile rose plant adsorbent removed 98.7% Pb^2+^ at a pH of 8.5 from the initial concentration of 5–40 mg/L (Abdel-Ghani and El-Chaghaby 2007). The Viscum album leaves had maximum adsorption capacity and maximum removal efficiency of 769.2 mg/g and 92.2%, respectively (Erenturk and Malkoc 2007). Several forest waste-based adsorbents have shown outstanding performances, which deserve further research for a more comprehensive application. Also, the toxicity of the adsorbents needs to be assessed prior to application for drinking water.

Several biotechnologies showed excellent removal efficiency (Table 6). The alginate encapsulated biosorbents (i.e., Agrobacterium fabrum) could be regenerated for reuse following desorption of the adsorbed Pb^2+^ ions. The maximum removal efficiency of the encapsulated Agrobacterium fabrum was 85%. The adsorbents were used repeatedly up to five times without affecting the adsorption capacity (Tiwari et al. 2017). The Chlorella Vulgaris adsorbents showed a removal efficiency of 99.4% (Goher et al. 2016). Up to 95% of Pb^2+^ were desorbed from alginate beads using citric acid, which were reused at almost a similar efficiency (Goher et al. 2016). The maximum adsorption capacity of phosphate-modified baker’s yeast was 92 mg/g (Liu et al. 2018). Although the biotechnologies showed very good to excellent efficiency, their application in the drinking water systems is discouraged as the effects of the biotechnologies are yet to be better understood. The performances of promising low-cost adsorbents are shown in Table 7.

**Fig. 2:**
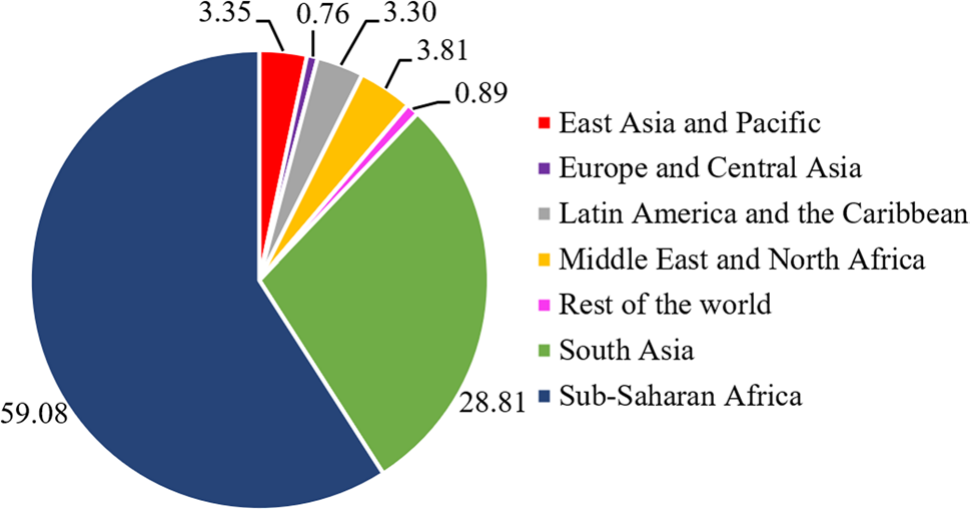
Regional share of people living below poverty line (%)

Lead ions in drinking water have been a historical issue as the leaded pipes were used in the water distribution systems (WDS), where lead was used to solder iron and copper pipes (Korshina and Liu 2019; WHO 2014). Recently, the presence of copper, chlorine, and lead in drinking water caused eight outbreaks (Brunkard et al. 2011; CDC 2013). Although many national and international standards and certification programs are in place to control lead contamination in drinking water, lead-containing pipes and fittings are still used (Dignam et al. 2019). In addition, the presence of lead in groundwater and surface water is an issue in low-income and developing countries. Globally, approximately 689 million people are living below the poverty line, whose income is US$1.90 or less a day (Aguilar et al. 2020). Besides, the COVID-19 pandemic is likely to force another 88–115 million people to live below the poverty, which might be increased to 150 million by 2021 (World Bank 2020a). In Sub-Saharan Africa, 433.4 million people were living below the poverty line (World Bank 2018). In South Asia, 211.3 million people were living below the poverty line (World Bank 2020b). The regional distribution of the populations living below the poverty line is shown in Fig. 2. Most of these people live in rural areas and cannot afford bottled water or advanced water treatment methods (Chowdhury et al. 2016). These populations mainly depend on groundwater and surface water for drinking and other household activities, whereas the sources might have higher lead levels than the acceptable values (Chowdhury et al. 2016).

The concentrations of lead in groundwater and surface water from several regions in Bangladesh were reported to be in the ranges of 0.04–1167 and 0–230 µg/L, respectively (Bhuiyan et al. 2015; Hasan et al. 2019). The average lead concentrations in shallow and deep tube wells were 1167 and 1120 µg/L, respectively (Mostafa et al. 2017). In India’s Hindon river, the mean lead concentration was 258 µg/L (30.1–902.1 µg/L) (Suthar et al. 2009). The mean lead concentration in groundwater of Darrang district, Assam, was 127.2 µg/L (40–350 µg/L) (Borah et al. 2010). The lead concentrations in the surface water and groundwater in Abakaliki, southeast Nigeria were 0–11,400 and 0–38,000 µg/L, respectively (Obasi and Akudinobi 2020). There is a need to develop low-cost technologies for individual and community-level applications to address these problems. The processed solid residue of olive mill products removed lead ions effectively, which is a costless agro-industrial byproduct (Tzamaloukas et al. 2021). Ahmedna et al. (2004) used the acid-activated almond shell-based carbon with steam-activated pecan shell in the point-of-use (PoU) filter to remove Cu^2+^, Pb^2+^, and Zn^2+^. The filter removed nearly 100% of lead ions. The average cost of the filter was estimated to be less than US$ 1.0 (Chowdhury et al. 2016). The maximum uptake capacity of dry protonated alginate beads was 339 mg/g (Lagoa et al. 2007). The maximum uptake capacity of sodium alginate-graft-poly (methyl methacrylate) beads was 526 mg/g (Salisu et al. 2016). The findings indicated that a large number of activated carbons could be developed at a low cost using the discarded waste and/or byproducts following further research. In addition, following the investigation on toxicity, a number of low-cost adsorbents can be used in the PoU filter to remove lead ions from drinking water.

## Post-adsorption management

The used adsorbents are likely to produce lead-containing sludge, which needs to be managed effectively. The used adsorbents can be controlled by regeneration, recycling, reuse, and safe disposal. The desorption process can do regeneration of the adsorbents. The desorption process uses acid (sulfuric acid, hydrochloric acid, nitric acid), base (sodium hydroxide), or salt (sodium chloride, ammonium chloride) as desorbing agents (Hamad and Idrus 2022). After a few adsorption–desorption cycles, the efficiency of the adsorbent decreases (Zhang et al. 2020a, b). However, several studies showed that the adsorbents could be regenerated without significantly reducing efficiency. Gupta and Rastogi (2008) used cyanobacterium Nostoc muscorum biomass, and the biosorbents were regenerated using HNO_3_ and EDTA. The regenerated biosorbent was used for five cycles without affecting the biosorption capacity. Katsou et al. (2011) used natural zeolite to adsorb lead and zinc. The adsorbent was regenerated using KCl, and the desorption efficiency was 98.5%. When there is no significant desorption, the adsorbent should be disposed of safely. The lead-containing adsorbents can be stabilized and/or solidified prior to landfill disposal (Hamad and Idrus 2022). The adsorbents can also be used as ingredients in the production of ceramic materials. Ceramic products may aid in the prevention of the leaching of heavy metals. For example, iron oxide nanoparticles were disposed of by immobilizing inside the phosphoric glass matrix (Majumder et al. 2019). In addition, the adsorbents can be used in the construction industry to form brick (Avinash and Murugesan 2019) or as a filler material in the cement industry (Saikia and Goswamee 2019).

## Future research

To date, different groups of adsorbents have been developed and applied to remove lead ions from water and wastewater. Significant success has been achieved in research and understanding lead contamination and its possible effects on humans. However, the development and application of low-cost adsorbents are still limited. The advanced technologies are often beyond the capacity of the low-income populations around the globe (Chowdhury et al. 2016). To develop low-cost technologies for low-income people, future research is warranted. The following studies should be carried out for developing low-cost adsorbents to remove lead ions from water and wastewater efficiently:

- The adsorbents are likely to produce large amounts of lead-containing sludge, which must be disposed of safely. Few past studies have reported the cementation techniques in which the lead-containing sludge is hardened and disposed of safely beyond the reach of water sources. Future studies may further assess this disposal technique's feasibility and economic benefits.
- The adsorbents developed to date were primarily used for wastewater treatment. As such, the effects of these adsorbents on humans were not given much attention. Future research is needed to assess the adsorbents' toxicity prior to their applications for the surface water, groundwater, and drinking water systems.
- Natural clay materials such as bentonite and zeolite, low-cost and widely available, showed excellent performances in treating lead-containing wastewater. Further investigation of these materials is needed in context to toxicity.
- Several industrial byproducts (i.e., steel slag, steel dust, fly ash, waste beer yeast, sawdust) showed promising results in removing lead ions from an aqueous solution. These materials are low-cost, and some of these can be obtained free of cost (e.g., waste beer yeast). Further investigation on these materials is needed to develop a low-cost adsorbent with high efficiency.
- Several agricultural wastes (i.e., dried tea leaves biomass, rice husk), forest waste (i.e., pinus pinaster, pinus elliottii bark), and industrial by-products (i.e., sawdust) based adsorbents showed very good to excellent performances in removing lead ions from wastewater. The raw materials of these adsorbents were cheap and likely to be environmentally friendly. These materials need further investigation to develop discharge filtration techniques for removing the lead ions. The raw materials can be tested for toxicity before application to drinking water.
- The bacterial biosorptions of heavy metals are likely to be the inexpensive technologies in removing heavy metals from aqueous solutions (Aryal 2021). Although several fungi (i.e., Aspergillus niger, Chlorella Vulgaris) have been used as biosorbents in the lab to remove lead ions from an aqueous solution, there are limited large-scale applications. Future study is needed to apply bacterial biosorbents for lead removal, which may help to develop low-cost, effective commercial biosorbents.
- Regeneration of adsorbents is an important technique to reduce the cost. The encapsulated biomass showed regeneration capability. For example, 95% of Pb^2+^ were desorbed from the immobilized Chlorella Vulgaris alginate beads. The encapsulated Agrobacterium fabrum was used for five consecutive cycles without reducing its adsorption capacity. There are limited studies on alginate-based biotechnologies in removing lead ions from water and wastewater. Future research is needed in this direction.
- Biosorbents are often used after modification using acid, base, or heat. Studies should be carried out to determine the minimum requirements of acid, base, or heat, which is likely to reduce the toxicity of the adsorbents and the cost. The modeling, regeneration, and immobilization of biosorbents deserve further investigation.

## Conclusions

Heavy metals, particularly lead in the aquatic environment, have become an issue due to their toxicity and long-term health implications. This review investigated the technologies for lead ion removal from water and wastewater, focusing on low-cost adsorbents. Different technologies are available to remove lead ions from domestic and industrial wastewater. Adsorption has been chosen as the most suitable technology due to its low cost, easy to use, and excellent removal efficiency. The different groups of adsorbents were investigated and compared. The advantages and disadvantages of these adsorbents were highlighted. The adsorption capacity and removal efficiency of the adsorbents were in the ranges of 0.7–2079 mg/g and 13.6–100%, respectively. Among the low-cost adsorbents, the adsorption capacity of carboxylated jute stick activated carbon, rice husk nanocomposite, Viscum album leaves, and activated aloji clay was 2079 mg/g, 1665 mg/g, 769.2 mg/g, and 333.3 mg/g, respectively. The corresponding removal efficiencies were 99.8%, 96.8%, 92.2%, and 97.3%, respectively. Future research directions were identified for developing and applying low-cost and easy-to-use adsorbents for removing lead ions from water and wastewater.
